# Genome sequencing revealed the red-flower trait candidate gene of a peach landrace

**DOI:** 10.1093/hr/uhad210

**Published:** 2023-10-18

**Authors:** Ping Zhou, Siru Lei, Xiaodan Zhang, Yinghao Wang, Rui Guo, Shaobin Yan, Guang Jin, Xingtan Zhang

**Affiliations:** Fruit Research Institute, Fujian Academy of Agricultural Sciences, Fuzhou 350013, China; Research Centre for Engineering Technology of Fujian Deciduous Fruits, Fuzhou 350013, China; College of Life Sciences, Fujian Agriculture and Forestry University, Fuzhou 350002, China; Shenzhen Branch, Guangdong Laboratory of Lingnan Modern Agriculture, Genome Analysis Laboratory of the Ministry of Agriculture and Rural Affairs, Agricultural Genomics Institute at Shenzhen, Chinese Academy of Agricultural Sciences, Shenzhen 518120, China; Boyce Thompson Institute, Cornell University, Ithaca, NY 14853, USA; College of Life Sciences, Fujian Agriculture and Forestry University, Fuzhou 350002, China; Shenzhen Branch, Guangdong Laboratory of Lingnan Modern Agriculture, Genome Analysis Laboratory of the Ministry of Agriculture and Rural Affairs, Agricultural Genomics Institute at Shenzhen, Chinese Academy of Agricultural Sciences, Shenzhen 518120, China; Fruit Research Institute, Fujian Academy of Agricultural Sciences, Fuzhou 350013, China; Research Centre for Engineering Technology of Fujian Deciduous Fruits, Fuzhou 350013, China; Fruit Research Institute, Fujian Academy of Agricultural Sciences, Fuzhou 350013, China; Research Centre for Engineering Technology of Fujian Deciduous Fruits, Fuzhou 350013, China; Fruit Research Institute, Fujian Academy of Agricultural Sciences, Fuzhou 350013, China; Research Centre for Engineering Technology of Fujian Deciduous Fruits, Fuzhou 350013, China; Shenzhen Branch, Guangdong Laboratory of Lingnan Modern Agriculture, Genome Analysis Laboratory of the Ministry of Agriculture and Rural Affairs, Agricultural Genomics Institute at Shenzhen, Chinese Academy of Agricultural Sciences, Shenzhen 518120, China

## Abstract

Peach (*Prunus persica*) is an economically important fruit crop globally and an excellent material for genomic studies. While considerable progress has been made in unveiling trait-associated genes within cultivars and wild relatives, certain novel genes controlling valuable traits in peach landraces, such as the red-flowering gene, remained unclear. In this study, we sequenced and assembled the diploid genome of the red-flower landrace ‘Yingzui’ (abbreviated as ‘RedY’). Multi-omics profiling of red petals of ‘RedY’ revealed the intensified red coloration associated with anthocyanins accumulation and concurrent decline in flavonols. This phenomenon is likely attributed to a natural variant of *Flavonol Synthase* (*FLS*) harboring a 9-bp exonic insertion. Intriguingly, the homozygous allelic configurations of this *FLS* variant were only observed in red-flowered peaches. Furthermore, the 9-bp sequence variation tightly associated with pink/red petal color in genome-wide association studies (GWAS) of collected peach germplasm resources. Functional analyses of the FLS variant, purified from procaryotic expression system, demonstrated its diminished enzymatic activity in flavonols biosynthesis, impeccably aligning with the cardinal trait of red flowers. Therefore, the natural *FLS* variant was proposed as the best candidate gene for red-flowering trait in peach. The pioneering unveiling of the red-flowered peach genome, coupled with the identification of the candidate gene, expanded the knowledge boundaries of the genetic basis of peach traits and provided valuable insights for future peach breeding efforts.

## Introduction

Peach originated in China [1]. The divergence event of peach species occurred during serial uplifts of the Tibetan plateau 2.47 million years ago (Mya), fitting well to a 2.6-million-year-old peach endocarp fossil evidence discovered in southwest China [6]. The favorable peaches were early selected by the ancients at least 7500 years ago in South China [2, 3]. Then, domesticated peach resources were introduced westward to Europe, America, and Africa, eastward to East Asia, and southward to Southeast Asia via intercontinental trade and migration from China [4, 5]. Evolutionarily speaking, the longstanding peach selection and domestication in China brought much more diversity in domesticates and landraces due to diverse climates and topographies [6]. The extensive climatic gradients and topographic changes resulted in at least seven different peach ecotypes that exhibited adaptive evolution under multiple selection pressures in China [7]. Comparative studies of these peach landraces are conducive to unveiling the genetic basis underlying novel traits in modern peach cultivars using genomic approaches [1, 7–19]. However, few superior landraces have been fully studied at the genomic level. Genomic analyses of peach landraces would facilitate the target loci selection and gene pyramid of peach breeding.

Peach landrace ‘Yingzui’ (non-melting flesh) was originally grown in southern China. The native ‘Yingzui’ population often consists of two types of individuals, including the late-ripening, red-flower ‘Yingzui’ (abbreviated as ‘RedY’) and the early-ripening, pink-flower ‘Yingzui’ (abbreviated as ‘PinkY’). Both ‘RedY’ and ‘PinkY’ show the morphology with pronouncedly accentuated tip and scarce epicarp pigmentation in their mature peach fruits, which resemble wild peaches of southern China [4]. Notably, the red petals coloration, usually displaying in ornamental peaches, exists in ‘RedY’, which is an edible peach landrace. Obviously, red peach flowers provide a fascinating color preference to attract tourists, comparing to pink peach blossom in most commercially freshly eatable peach. Why the intensified red coloration appeared in red-flower peach petals remained unclear.

The earliest genetic study of flower color variants in peaches could date back to the 1940s [20], revealing petal colorations were determined by two independent qualitative genetic loci R and W for pink/red (R_/rr) and white-flower (ww) phenotypes [21]. It is widely acknowledged nowadays that the difference in petal pigmentation results from anthocyanin content [21–23]. The defect of anthocyanin transport and storage conferred an anthocyanin-deficient phenotype characterized in white flowers or variegated flowers [24, 25]. A dramatic increase in anthocyanin content was identified in red flowers compared to pink flowers, suggesting the red-flower trait gene was likely involved in the regulation of anthocyanin biosynthesis and accumulation [22, 23, 26]. In flavonoids biosynthetic pathway, Coumaroyl-CoA substrate was initially converted to dihydroflavonol intermediates by Chalcone Synthase (CHS), Chalcone Isomerase (CHI), and Flavanone 3-hydroxylase (F3H). Then dihydroflavonol intermediates were partially catalyzed to yield anthocyanidins by Dihydroflavonol Reductase (DFR) and Leucoanthocyanidin Dioxygenase (LDOX) while the remaining intermediates were parallelly used for flavonol syntheses [27]. These biosynthetic genes mentioned above could be transcriptionally regulated via a variety of MYBs, bHLHs, and WD40s complex proteins (MBWs) in *Prunus persica* [28–38]. Thus, transcriptional changes of regulatory and biosynthetic genes (such as *PpMYB9*, *PpMYB10.2*, *PpMYB108*, *PpPeace*, *PpMYBPA1*, *PpMYb17*, *PpMYb18*, *PpMYb19*, *PpMYb20*, and *PpLDOX*) were considered to be vital for diversity of peach flowers in their differentiated anthocyanins content levels [22, 26, 39–41]. However, to date, the crucial determinants of red-flower trait have not been proposed and verified in red-flowered peach germplasms, which hinders the exploitation and utilization of red-flower peaches.

To uncover the genetic basis of red-flower coloration trait in peaches, we assembled a chromosome-scale diploid genome of red-flower peach landrace ‘RedY’ and identified a red-flower determining candidate gene supported by genotype identification and genome-wide association studies. This candidate was a natural variant of Flavonol Synthase (FLS), which was associated with accumulated anthocyanins and declined flavonols caused by its diminished catalytic activity in red-flower peaches. Our findings provide valuable insights into the genomic characteristics and petal coloration mechanism of peaches, advancing the understanding in this area of research.

## Results

### Chromosome-level and haplotype-resolved genome assembly and characteristic analyses

**Figure 1:**
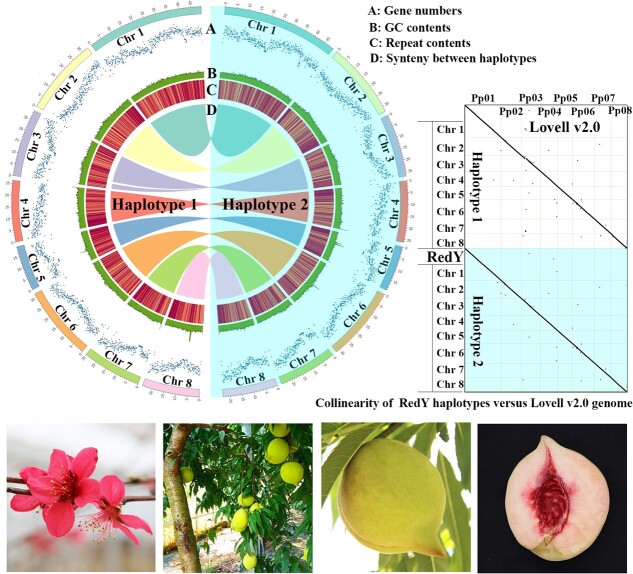
Features of *Prunus persica* ‘RedY’ genome.

The high-quality chromosome-level genome of peach landrace ‘RedY’ was assembled based on PacBio high fidelity (HiFi) sequencing and High-throughput chromatin conformation capture (Hi-C) sequencing reads (Table 1, Fig. 1). A total of 15-Gb of PacBio HiFi reads, representing the 60-fold sequencing coverage, were assembled into two haplotype-resolved contig-level genomes which consisted of 145 and 122 contigs with the N50 value of 16.7 Mb and 25 Mb, respectively. With 67-Gb Hi-C data, 145 and 122 contigs were anchored into eight chromosomes, resulting in two final haplotype-resolved assemblies with a total length of 242.77 Mb and 247.40 Mb, respectively. The anchor rate of both haploid assemblies was over 96%. We utilized Circos plotting to visualize chromosome-scale information on gene density, GC content and repetitive sequences density for each haplotype. Both haploid assemblies showed high collinearity with the extensively studied Lovell v2.0 peach genome (Fig. 1).

**Table 1:** Summary of de novo genome sequencing and assembly

**Statistic items**	**Numerical values**	
**Sequencing data**		
PacBio Sequel II sequencing		
Raw data (Gb)	14.995	
Sequencing depth (×)	60	
Reads average length (bp)	14 852	
Reads max length	63 256	
Reads *N*_50_ length (bp)	15 374	
Hi-C sequencing		
Clean data (Gb)	67.05	
Sequencing depth (×)	268	
**Haplotype-resolved assembly**	**Haplotype 1**	**Haplotype 2**
Contigs number	145	122
Contigs *N*_50_ / *N*_90_ length (bp)	16 726 405 / 3 800 866	25 067 346 / 6 848 511
Chromosomes number / length (bp)	8 / 242 771 195	8 / 247 406 482
Chromosomes *N*_50_ / *N*_90_ length (bp)	26 992 111 / 19 518 747	29 041 490 / 19 386 848
Chromosomes anchor rate (%)	96.30	96.14
Chromosomes LTR assembly index	25.51	26.09
BUSCO completeness of assembly (%)	98.5	98.8
GC content	37.95	38.09

Overall, 29 249 and 29 431 putative genes were annotated for two haplotypes using *de novo* predication, transcriptome mapping, and homology search approaches. More than 93.87% Illumina RNA-Seq clean reads that had been used for gene annotation could be mapped to the diploid genome (Table S1, see online supplementary material). BUSCO completeness of the annotation of RedY genome (comprised two haplotypes) was estimated to be 99.1%. Our analysis revealed that repetitive sequences accounted for 36.48% of the assembled genome, with long terminal repeat retrotransposon (LTR-RT) being the most abundant, accounting for 53.5% of the total repetitive sequences. Of these, Ty1/Copia and Ty3/Gypsy, the two major clades of LTR-RT elements, covered 4.87% and 7.01% of the diploid genome, and other DNA transposons took up 18.02% of the genome sequence (Table S2, see online supplementary material). Kimura distances indicated a burst of LTR-RT activity ~6.75 Mya (Fig. S1, see online supplementary material).

### Comparative analyses revealed a differential content changes of anthocyanins and flavonols in red flowers of ‘RedY’ contrasting to pink flowers of ‘PinkY’

**Figure 2:**
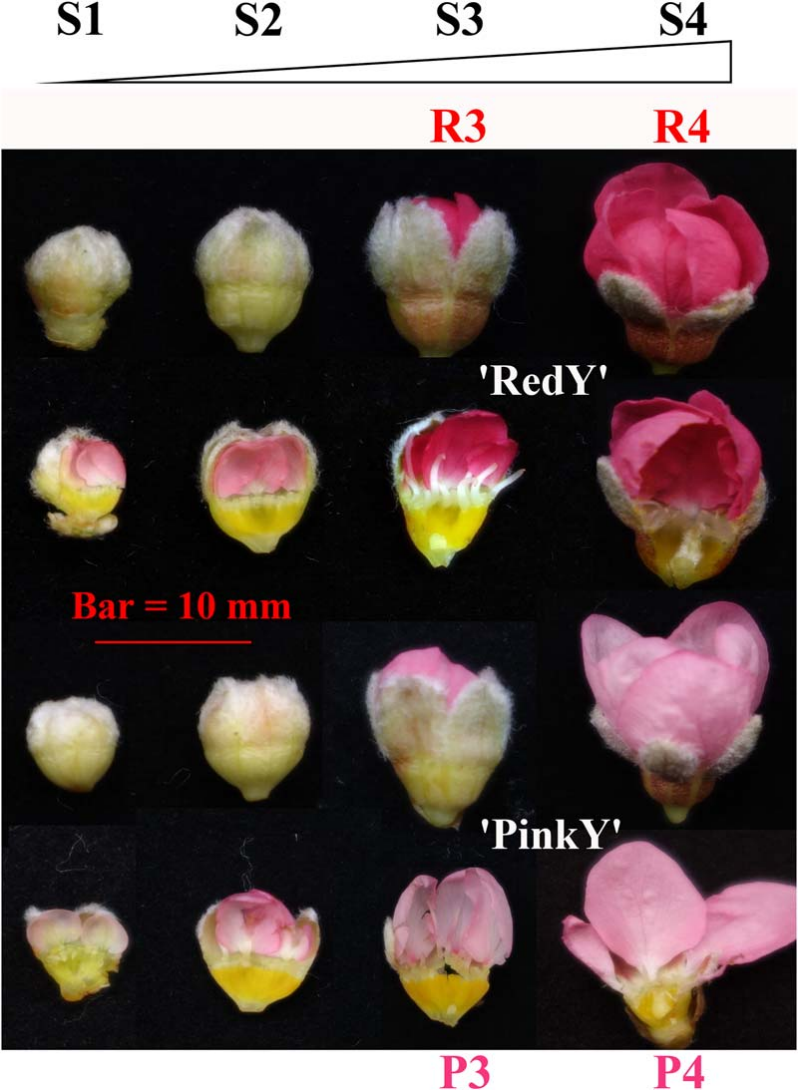
The morphologic characteristics and developmental states of floral samples of ‘RedY’ and ‘PinkY’.

With the assembly of the first genome of red-flower peach, the cause of intensified red coloration of ‘RedY’ flowers has gained special attention. To further clarify the petal coloring process, four types of floral samples (S1–S4), representing differential morphological characteristics and developmental states, were collected for anatomic observation (Fig. 2) from ‘RedY’ and ‘PinkY’ (the pink-flower counterpart in native ‘Yingzui’ peach population). The light colorations in the parallel samples at the S1 stage before petals sprouted out of the flower bud were discovered. Then the primary petal pigmentation process began from tips and margins, until it spread to the whole petal. The full pigmentation of ‘RedY’ petals appeared earlier and was more intensive than that of the ‘PinkY’ counterparts. At the stages of S3 (ballooning) and S4 (bursting into full flower), a distinct difference in red coloration intensity was observed between ‘RedY’ and ‘PinkY’ petals; therefore, those color-contrasting petal samples (namely R3 and P3, R4 and P4) were selected for pigment substance determination.

**Figure 3:**
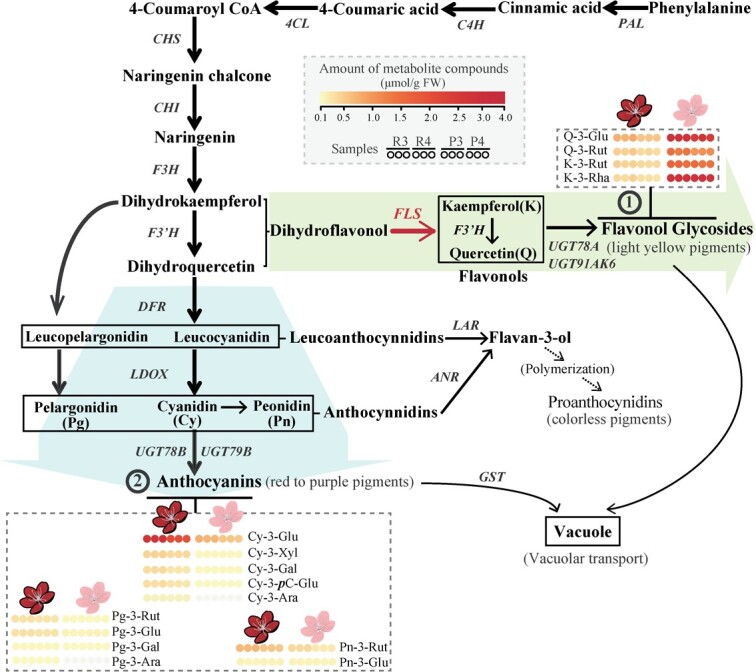
The main anthocyanins and flavonol glycosides contents in the corresponding biosynthetic pathway. In this diagram, ①② represent two important branches of pigmented products in flavonoid biosynthetic pathway. 4CL, 4-Coumarate: CoA ligase; ANR, anthocyanidin reductase; C4H, cinnamate 4-hydroxylase; CHI, chalcone isomerase; CHS, chalcone synthase; DFR, dihydroflavonol reductase; F3H, flavanone 3-hydroxylase; F3’H, flavonoid 3′-hydroxylase; FLS, flavonol synthase; GST, glutathione S-transferase; LAR, leucoanthocyanidin reductase; LDOX, leucoanthocyanidin dioxygenase; PAL, phenylalanine ammonia-lyase; UGTs, glycosyltransferases.

Previous studies indicated that discrepancies in the coloration of peach petal were associated with changes in levels of anthocyanin content [21–23]. Additionally, the competitive substrate-use for parallel flavonol syntheses alleviated the pigmentation of petals [23]. Thus, we determined the content of main anthocyanin and flavonol components from color-contrasting samples of ‘RedY’ and ‘PinkY’, including R3, R4, P3 and P4 (Fig.3; Table S3, Note S1, see online supplementary material). The amounts of anthocyanin metabolites of ‘RedY’ were significantly higher than that of ‘PinkY’ (R3 vs P3, R4 vs P4). Importantly, according to the calculation results, the abundances of important Cy-3-Glu, which account for over half of the anthocyanin metabolites and is far more than other anthocyanin derivatives, in ‘RedY’ was 4-fold higher compared with ‘PinkY’. In contrast, the flavonol content of ‘RedY’ showed a decline compared with that of ‘PinkY’. The metabolomics results suggested that a strengthening anthocyanin accumulation accompanied by a massive decline in flavonols production is associated with the intensified red coloration of ‘RedY’ petals.

### Screening of gene(s) related to abundance changes of anthocyanin and flavonol composition

In *P. persica*, red pigmentation of tissues has been intensively studied [28–39], and most critical genes for anthocyanin and flavonol biosyntheses, coupled with their important regulatory transcription factors, were identified from their complex gene families based on omics evidences and experimental validation of the involvement of red pigmentation [22, 26, 33, 34, 36, 38, 39]. Consequently, we directed our focus towards evaluating the transcriptional dynamics of 53 genes that corresponded to the above-reported anthocyanin/flavonol-related biosynthetic or modifying genes and specific MBWs complex regulators (53 genes information listed Table S10, see Materials and Methods section and the online supplementary material). We separately aligned transcriptomic reads of collected petal samples against the ‘RedY’ genome (corresponding sequencing and mapping statistics listed in Table S4, see online supplementary material) and calculated gene expressional levels. The investigation result of selected genes suggested that, in red-pink petals comparisons (R3 vs. P3 and R4 vs. P4), most reported anthocyanin/flavonol-related biosynthetic genes (*PpPAL*, *PpC4H*, *Pp4CL*, *PpCHS*, *PpCHI*, *PpF3H*, *PpF3’H*, *PpFLS*, *PpDFR*, *PpLDOX*/*PpANS*) and transcriptional regulator (*PpbHLH3*, *PpWD40*, *PpHYH*, *PpHY5*, *PpCOPs*, *PpMYB10s*, *PpMYBPA1*, *PpMYBF1*, *Peace*, *PpMYB17*–*20* and *PpMYB108*) did not show higher transcriptional expression levels in red petals contrasting to pink petals (Fig. S2, see online supplementary material). Moreover, these selected genes did not display consistent change trends in both pink-red petals comparisons. The expressional change tendencies of specific genes were also confirmed by the parallel qRT-PCR verification (Fig. S3, see online supplementary material), which showed their consistency with comparative transcriptomic analyses.

In addition to the preliminary comparative results of transcriptional dynamics, the analyses of correlations between anthocyanins/flavonols amounts and regulatory *MBWs* expressional levels in all collected samples also showed that the absolute values of the Pearson correlation coefficient were lower than 0.7, which inferred insufficient relevances (Table S5, see online supplementary material). Combined with both results, we perceived that the expressional changes of selected structural genes and *MBWs* were unlikely to explain the anthocyanin/flavonol content changes in red-pink petal comparison.

Subsequently, we performed the comparative transcriptome analyses of color-contrasting samples, resulting in 969 and 1336 differentially expressed genes (DEGs) respectively in R3 vs. P3 and R4 vs. P4 comparisons. Twenty-four upregulated and 82 downregulated DEGs commonly existed in red petals when comparing to the pink counterpart (Fig. S4, see online supplementary material). Gene Ontology and KEGG annotations of 106 common DEGs had not found any putative genes that may associate to the anthocyanin/flavonol pathway (Table S6, see online supplementary material). Monoterpenoid biosynthesis, ascorbate and aldarate metabolism were the significantly enriched metabolic pathway by analysing common DEGs (Table S7, see online supplementary material). Summarily, no evidences could connect the gene expression shift of anthocyanin/flavonol-related structural genes to anthocyanin/flavonol content change.

**Figure 4:**
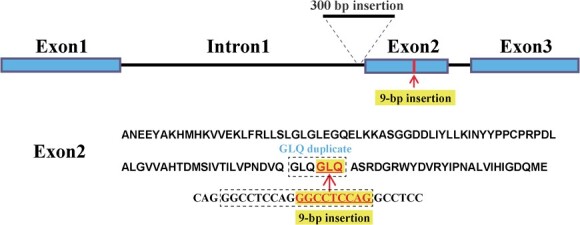
The nucleotide and deduced amino acid sequences of ‘RedY’-specific FLS variant. A 9-bp insertion made a novel GLQ (Gly-Leu-Gln) duplicate in ‘RedY’-sourced Flavonol Synthetase (peach05454).

Other than transcriptional regulation, genetic variance of structural or regulatory genes may affect target metabolite production or accumulation. After checking the results of aligning ‘RedY’ annotated genes/proteins with the 53 aforementioned anthocyanin/flavonol-related genes/proteins retrieved from previous peach anthocyanin/flavonol studies [22, 26, 33, 34, 36, 39], a novel ‘RedY’-specific *FLS* (*Flavonol Synthetase*) variant with 9-bp insertion in its second exon and a 300-bp insertion in the first intron was found and characterized, whereas other functional genes/proteins showed high similarities in both nucleotide and deduced amino acid sequences. The *FLS* variant is shown schematically in Fig. 4. Blasting this FLS variant in the non-redundant protein database (NCBI host), no similar amino acid insertion was identified in peach or other species, suggesting the insertion of GLQ (Gly-Leu-Gln, encoding by 9-bp exonic insert) formed a GLQ duplicate which gives rise to a scarce FLS variant in the plant kingdom (Fig. S5, see online supplementary material). Considering that conserved GLQ motifs were present in all investigated FLS proteins, implicating its possible important role, the GLQ duplicate variance of FLS may have impacted enzymatic activity. We preliminarily selected the *FLS* variant as the candidate gene responsible for anthocyanin or flavonol abundance changes.

### Variant validation and genotyping of FLS in pink- and red-flower peaches

Comparing to the previously studied FLS gene (Prupe.1G502800, Lovell v2.1), two distinct insertions occurred in current FLS gene variant, one in intron1 and the other in exon2 (details of sequence variance are shown in Fig.S6, see online supplementary material). Our validation results of 168 pink-flower and 23 red-flower peaches showed that a 300-bp intronic insertion was present in some pink-flower and all red-flower peaches. This observation renders 300-bp intronic insertion less likely to be the candidate responsible for red-flower traits. On the other hand, a predominant presence of 9-bp exonic insertion was observed in all red-flower peaches, suggesting the potential association of the 9-bp exonic insert with the red-flower characteristic. The concrete results of insertion sequence validation are summarized in Table S8 (see online supplementary material).

**Figure 5:**
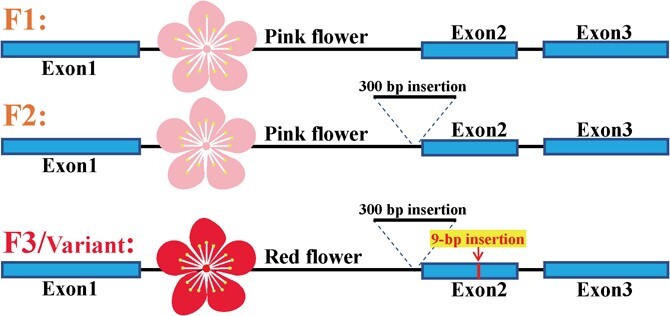
Three *FLS* alleles detected in pink-flower and red-flower peaches. F1, F2, F3 were three types of *FLS* alleles. Comparing to F1, F2 harbors 300-bp intronic insertion while F3 (referring to *FLS* variant) contains both 300-bp intronic and 9-bp exonic insertion. F1 and F2 can be identified in pink-flower peaches, and F3 predominantly existed in red-flower peaches.

According to the validation results of two insertions among collected peaches, we identified at least three *FLS* alleles (F1, F2, and F3). Of these, F3 allele/variant contained both two insertions while F2 allele had 300-bp intronic insertions and F1 allele was without insertion (Fig. 5). Significantly, the homozygous genotype harboring 9-bp exonic insertion (F3F3) was exclusively found in red-flower peaches. We genotyped 169 pink-flower and 23 red-flower peaches with *FLS* alleles (genotyping results also listed in Table S8, see online supplementary material), confirming that the F3F3 allelic genotype was only present in red-flower peaches while other genotypes appeared in pink-flower peaches. This suggests that the genotype F3F3 is specifically associated with the red-flower trait.

### FLS variant is the best candidate gene for red-flower trait in peach

**Figure 6:**
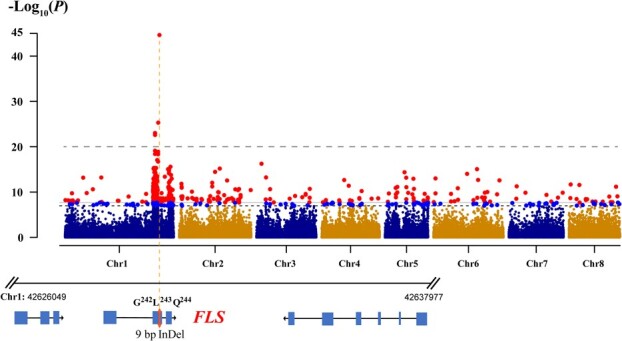
Manhattan plot for GWAS of the petal colors. The 9-bp InDel variance of *FLS* on chromosome 1 is tightly associated with pink-red petal color.

The red-flower phenotype has been previously reported as a qualitative trait determined by a recessive locus [21]. Using ‘RedY’ high-quality haploid genome and peach re-sequencing data, the GWAS analysis was performed to investigate the genome-wide associations between SVs (including SNP and InDel) and petal-color phenotypics. Considered the threshold line of *P* < 1E-20, only significant association peaks were found on chromosome 1. The top associated SV, most strongly related to red-flower phenotype, was mapping in exon 2 of *FLS* (Fig. 6). It was a 9-bp InDel variance of *FLS* sequence, resulting in the presence/absence of GLQ (Gly-Leu-Gln) in the amino acid sequence, which is consistent with our present findings. Besides this mapped *FLS* (*peach05454*, RedY), other neighboring high-associated SVs of association peak were distributed in intergenic regions, and SV adjacent genes were irrelevant to the anthocyanin/flavonol pathway (Table S9, see online supplementary material). Thus, based on the highest association between red-flower phenotype and sequence variance, certain *FLS* (*peach05454*, RedY) was identified as the best candidate gene determining the red-flower trait.

Notably, in addition to *peach05454*, three putative proteins, coded by tandem duplication genes (*peach05450*, *peach05451*, and *peach05453*), displayed 49% to 59% similarity to functional AtFLS1 (coding by *Arabidopsis thaliana AT5G08640*) (Fig. S7a, see online supplementary material). However, the expression of these three tandem genes was scarcely detected in roots, young and mature leaves, flowers, exocarps and mesocarps (Fig.S7b, see online supplementary material), suggesting that *peach05450*, *peach05451,* or *peach05453* were silenced or suppressed, with *peach05454* being the predominant gene exhibiting the certain FLS function. Therefore, considering the genomic location of the InDel variance in the GWAS and its major gene status, we proposed that the *FLS* variant was likely contributing to the monogenic inheritance of red-flower trait.

### *FLS* prokaryotic expression and functional studies

**Figure 7:**
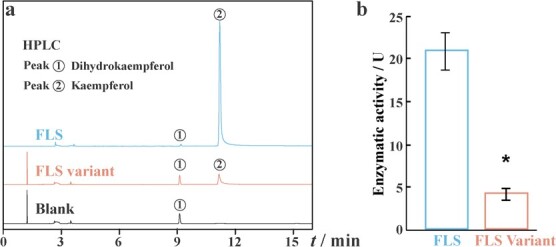
HPLC analysis of FLS catalytic reaction (**a**) and their corresponding enzymatic activities (**b**). * indicates the significant difference (Student *t*-test, *n* = 3).

As both F1 and F2 allele *FLS* were transcribed and translated into identical FLS proteins, regardless of the presence of 300-bp intronic insertion variance, we investigated and compared the enzymic activities between FLS (encoded by F1/F2 allele) and FLS variants (encoded by F3 allele). The results demonstrated the reduction in catalytic activity of the FLS variant, purified from the *Escherichia* expression system in converting dihydrokaempferol to kaempferol. Impressively, enzymatic activity was decreased by 78.66% in FLS variant, from 20.62 to 4.14 (Fig. 7), due to a GLQ insertion in the protein sequence. Our findings collectively point towards the fact that the FLS variant’s presence significantly curtails its ability to facilitate the synthesis of flavonols. Consequently, this reduction in flavonol synthesis capacity potentially exerts an influence on the delicate equilibrium between flavonols and anthocyanins within the context of peach petals.

## Discussion

In our study, we assembled a chromosome-level genome of RedY, a superior peach landrace grown in South China. The genome assembly was based on high-accuracy long reads, which allows us to gain new insights of its genomic characteristics, especially in repetitive sequences. Our analyses of genomic sequence suggested that LTR-RTs, the most abundant repetitive elements, might contribute to the genome expansion for their recent amplification 6.75 million years ago (Mya). This LTR-RTs burst time of RedY genome was earlier than the *P. persica* evolutionary split from other wild peach relatives (2.47 ~ 3.8 Mya) [1, 42], suggesting that LTR-RTs burst was an ancient event likely occurred in the oldest peaches ancestor. Lack of recent large-scale bursts of retrotransposons may maintain the small and compact nuclear genome size of *P. persica*, considering two LTR-RTs insertion bursts (1.07 ~ 1.17 and 5.50 ~ 5.69 Mya) expand Malus spp. genomes to 652 ~ 668 Mb [43]. Also with this latest peach genome, we discovered and identified a *FLS* natural variant (*peach05454*, RedY) as the red-flower determining candidate gene. The findings, combining multi-omics and physiological detection evidences, supported the candidate likely resulting in the accumulated anthocyanins and declined flavonols due to its diminished catalytic activity in red-flower peaches, which improved our understanding of petal coloration mechanism in *P. persica*.

### FLS variant is the red-flower determining candidate gene in peaches

Earlier studies have linked the color of peach flower with the natural abundance of anthocyanin substances [21, 23, 25, 26]. Our results showed that not only anthocyanin but also flavonol, another type of flavonoid compound, were associated with peach petal coloration. Interestingly, we observed a negative correlation between anthocyanin and flavonol, as the increase of anthocyanidins coincided with a decrease of flavonol glycosides in peach red petals.

In the flavonoid biosynthetic pathway, dihydroflavonol compounds were converted into anthocyanidins (by DFR) or flavonols (by FLS). The competitive equilibrium of dihydroflavonol substrate utilized by DFR or FLS has mediated the proportionate diversion of early precursors, which further participate into anthocyanidins or flavonols branch pathways [23, 44–48]. This explains the phenomenon observed in our study, where anthocyanidins were increased and flavonols were decreased in red peach flowers.

Previous transcriptomic analyses have shown that MYB transcription factors play a role in regulating anthocyanin-proanthocyanin or flavonol compound biosynthetic genes [22, 26, 32, 34, 37, 40]. Heterologous validations using transient expression systems have observed that *PpMYB10.2*, *PpMYB9*, or *PpMYBPA1*, *PpPeace*, and *PpMYB7* could activate transcriptional expression of anthocyanin or proanthocyanin biosynthetic genes, respectively, while *PpMYB17–20* negatively regulated flavonoid pathway genes [32, 40]. Besides, *PpMYB108*, *PpMYBPA1, PpMYB15*, and *PpMYBF1* were involved in regulating anthocyanin and flavonol biosynthesis in peach [22, 26, 32, 34]. However, in our study, the majority of reported MYBs and flavonoid biosynthetic genes did not display significant transcriptional differences between the red and pink petals of peach with similar genetic background, when we analysed gene expression profiles of the same developmental stage samples. Some genes, such as *PpMYB10.2*, *PpMYB108*, and *PpPeace*, which were thought to play vital roles in peach petal coloration [22, 26, 34], showed lower transcriptional abundance in red petals compared to pink petals, suggesting these MYB factors might not be the major red-flowering determinant in peach ‘RedY’. Furthermore, the GWAS studies have not found MYB variants related to the red-flowering trait in peach germplasm. Consequently, these MYBs were more likely to be involved in orchestrating the multi-branching flavonoid synthesis at a series of delicate developmental processes if their expressions and effects were systematically considered at sequential development stages.

Fundamental catalytic enzymes exert more direct actions upon the production and accumulation of target metabolite, flavonols and anthocyanins in flavonoid biosynthetic pathway. According to contrast content changes of flavonols and anthocyanins between RedY and PinkY petals, the vital enzyme of flavonols production, FLS, which has an ability to affect the balance of flavonol and anthocyanin, was ranked in priority after flavonoid-related gene screening. The variance of FLS could be the candidate for determining red coloration in red-flowered peaches for the following reasons. First of all, FLS-led flavonol biosynthesis can affect DFR-led anthocyanins accumulation. There is a strong competition between flavonols and anthocyanins because they originated from the same intermediate substrates [49, 50]. The transcriptional disequilibrium between FLS and DFR dramatically influenced the accumulation of anthocyanins in flowers of seven species, thus exhibiting a wide variety of red coloration, from light to dark [23]. Furthermore, attenuating flavonol synthase by suppressing or silencing *FLS* expression could enhance the anthocyanidins accumulation in *Petunia*, *Nicotiana*, *Arabidopsis*, *Mimulus*, and *Malus* species [23, 44, 46–48, 51]. Two independent studies have shown that the red pigmentation of hypocotyl and cotyledons during germination in *A. thaliana* knockout mutant *Atfls1* was more than twice the anthocyanin pigment accumulation in wildtype plants [45, 46]. Besides, the *Atfls1* mutant seedlings significantly reduced flavonol glycoside content in addition to increased accumulation of anthocyanidins, which demonstrated the biosynthetic competitiveness between anthocyanidins and flavonols [45, 46]. Similarly, *FLS* antisense suppressed petunia and tobacco have intensified red flower pigmentation and concurrently reduced flavonol synthesis in petals [44]. Additionally, the anthocyanin contents of *MlFLS*-RNAi transform *Mimulus* plants were twice that of the wild-type in their petal lobe [51]. In the Rosaceae family, silencing endogenous *FLS* expression by Agrobacterium-meditated VIGS infiltration induced visible red pigmentation in infected crabapple leaves and apple peels [48]. These cases suggested that diminishing *FLS* effects can effectively promote anthocyanins accumulation.

In our study, we found that the *FLS* variant (*peach05454*) containing a 3-amino acid insert, derived from red-flowering peach ‘RedY’, had poor activity converting dihydroflavonol to flavonol products *in vitro*. The low activity of the FLS variant reduced competition from the flavonol branch, channeling more overlapping substrates—dihydroflavonol intermediates into anthocyanidins synthesis branch—and thus determining higher accumulation of anthocyanidins in red petals, contrasting with pale pink petals.

This *FLS* insertion variance was detected in all collected red-flower peaches and was identified as the most prominent monogenic locus for red flowering traits. To our knowledge, the *FLS* variant (*peach05454*, RedY) was the first gene successfully identified by GWAS analysis of red-flower trait. The absence of high-quality red-flower-peach genome and the difficulties in identifying short-sequence duplication insertion by short-reads alignment likely delayed the discovery of red-flower trait-related *FLS* variance. Besides, we noticed that PpMYB10.2, PpMYB9, and PpMYB108’s vital roles in anthocyanin accumulation were first reported in red-flowering peach cv. ‘Man Tian Hong’ and ‘Hong Shou Xing’ whereas both peaches harbor the novel *FLS* variant with 9-bp exonic insert.

It is also noteworthy that three genes showing similarity to the functional *AtFLS1* were adjacent to the *FLS* variant (peach05454). Even though tandem duplication genes exit in the genome, the expressional profiling across various tissues indicated that peach05454 was the major gene. This mirrors *A. thaliana*, where *AtFLS1* is the sole functional gene, and the other five *AtFLS* members were either silenced genes or pseudogenes [45, 46]. While only PpFLS (Prupe.1G502800*,* identical to peach05454 except the 9-bp exonic insertion) was selected for study as previously reported [23, 34], the functionality and evolutionary relationships of other three putative genes merit further exploration to understand their potential roles and reasons for being silenced or suppressed.

Taken together, more pieces of evidence combined with genomic, metabolic, and enzymic analyses have now proven that the *FLS* variant is the best candidate for red-flowering genetic determinant in peaches. We proposed that once the enzyme activity of FLS was dramatically decreased, dihydroflavonol substrates would be largely catalyzed and convert to anthocyanidins products in ‘RedY’ and other red-flower peaches, resulting in an apparent red-flowering trait.

### Combination of FLS alleles acts as critical genetic factor determining the differential red-coloration of peach petals

**Figure 8:**
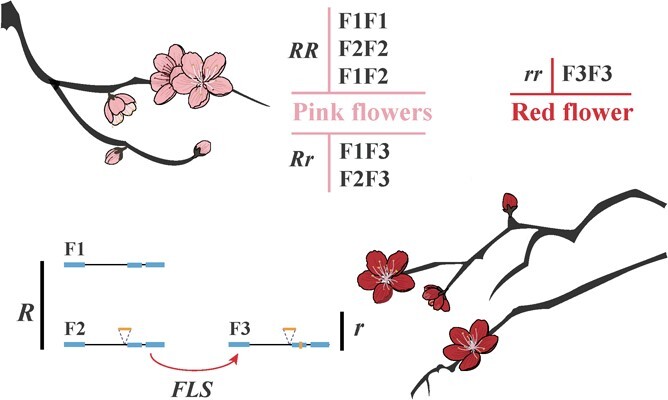
Genetic pattern and genetic variances of pink/red-flower determinant (*FLS*) in pink and red-flower peaches. F1, F2, and F3 are *FLS* alleles. F1 and F2 are identified to be *R* genetic factor, while F3 is *r*. Allele F3 may diverge from allele F2 due to the exonic insert. Genotype RR and Rr present pink-flower trait, and rr determines red-flower characteristic.

Our short-reads alignment revealed the presence of at least three *FLS* alleles, of which one variant (F3) was found in red-flower peaches, while the other two alleles (F1 and F2) were present in pink-flower peaches. We observed that red-flower peaches could be represented by the genotype F3F3. A previous study had reported that red/pink coloration of peach petals was controlled by a monogenic loci/factor *R* (where Rr or RR represents pink while rr represents red). Genetically speaking, the FLS proteins translated from alleles F1 and F2 were identical, as the intronic insertion could not have affected transcription splicing and translation. Therefore, *FLS* alleles F1/2 could be considered as a genetic factor *X*, while allele F3 should be regarded as an allelic genetic factor *x*. For our investigation result of collected peaches, only homozygous state *xx* (F3F3) appeared in red-flower peaches, while homozygous state *XX* (containing F1F1, F2F2, and F1F2) and heterozygous *Xx* (F1F3 and F2F3) were observed in pink-flower peaches. We proposed that x (referring to F3) represents r and X (referring to alleles F1/2) represents R. Furthermore, a *FLS* allele harboring 9-bp exonic insert but lacking 300-bp intronic insert was not found in any collected peach germplasms. Therefore, it is more likely that allele F3 diverged from allele F2, as the sequence difference between F3 and F2 is minimal. Allele F3 may have arisen as a consequence of a 9-bp insert in exon2 of allele F2. Our findings and hypothesis provide a fundamental explanation for the Mendelian genetic pattern and vital genetic variances of pink/red-flower determinant (Fig. 8). The identified gene resource of *FLS* variant can be utilized in peach gene pyramid with the purpose of breeding new cultivars that meet both ornamental and edible requirements.

## Materials and methods

### Plant materials

The cultivated peach landrace ‘RedY’ was selected for sequencing and assembly to obtain a high-quality genome. This landrace was propagated by bud grafting onto Maotao rootstocks, and then all 4-year-old grafted plants were grown in Jian’ou Agricultural Experimental Farm (affiliated to Fruit Research Institute, Fujian Academy of Agricultural Sciences, China), with meticulous field management. Landrace ‘PinkY’ and other germplasm materials were also collected and maintained in the experimental farm, following the same procedures as ‘RedY’.

### Genomic DNA extraction and denovo sequencing

Large molecular weight and high-quality genomic DNA were extracted and purified from fresh young leaves of a single ‘RedY’ tree. A 15 Kb SMRTbell library was constructed using SMRTbell Template Prep Kit 1.0 (Pacific Biosciences, San Diego, CA, USA), following the recommendations of its manufacturer. All standard procedures, including DNA shearing, damage repair, fragment end ligation, and target size selection, were performed according to protocols. Subsequently, the PacBio Sequel II platform (Pacific Biosciences, San Diego, CA, USA) was used for the single-molecule real-time sequencing, which generated 1.009 million PacBio high fidelity (HiFi) reads.

High-throughput chromatin conformation capture (Hi-C) libraries were similarly created from the same young leaves. Briefly, cross-linked DNA was digested by DpnII (Ipswich, MA, USA), and then digested fragments were sequentially end-repaired, biotinylated, and ligation-circularized. Fragments within the size range of 300–700 bp were further captured and purified for DNA library sequencing. A total of 225.383 million paired-end reads (Q30 bases achieved 91.98%) of sequencing data were obtained from the Illumina sequencing platform (Illumina, San Diego, CA, USA).

The single-molecule real-time sequencing and Hi-C sequencing experiments were performed at Novogene (Beijing, China) and Biomarker Technologies (Beijing, China), respectively.

### Genome assembly

Genomic contig sequences were initially assembled by hifiasm (0.16.1-r375) [52] with HiFi reads. These contigs were subsequently anchored into chromosomes following Hi-C analyses and manually corrected results by Juicer v1.6 [53], 3D-DNA v180419 [54], and Juicebox v2.20.00 [55] adopting official guidelines or manuals. The continuity and integrity of the assembly results were substantially improved by allelic contigs ordering with ALLHiC assembler [56]. Genome integrity and completeness were firstly assessed by BUSCO [57] evaluation of evolutionarily conserved single-copy protein-coding sequences (BUSCO v5.2.2 and embryophyta_odb10 databases). Then, LTR_retriever v2.9.4 [58] was used to determine the quality of the genome assembly based on the ratio of complete LTR retrotransposon sequence to total LTR sequence length, LTR assembly index (LAI). And we also utilized jcvi [59] to plot the genome-wide chromosomal synteny comparison result of ‘RedY’ haploid genome and published Lovell 2.0 genome to validate new assemblies’ accuracy and reliability.

### Genome annotation, genome evaluation, and TE analyses

The gene annotation, gene prediction, and pseudogene screening were completed using GETA v2.5.4 (http://github.com/chenlianfu/geta, adopt the default settings and libraries) in combination with RNA-seq data from six tissues (young leaves, mature leaves, flowers, young root, epicarp and mesocarp of near-mature fruit). The total mapping rates of RNA-Seq reads to haplotype-resolved genome were estimated by HISAT2 v2.2.1 [60] through aligning clean reads (generated by filtering Illuminar raw reads using fastp v1.2.3 [61]) with assembled diploid genome and its annotation file following default parameters and the completeness of the gene annotation was assessed by BUSCO with the same database libraries mentioned above.

In addition, whole-genome annotation of repetitive sequences was obtained by Extensive de novo TE Annotator (EDTA) pipeline [62] with default settings. LTR retrotransposon burst dating was estimated using the previously reported method [63] based on earlier calculated base mutation rate of peach genome (7.7 × 10^−9^ substitutions per site per year) [13].

### Petals anthocyanins determination and gene expression analyses in landrace ‘RedY’ and ‘PinkY’

To obtain ideal experimental materials for comparative study while minimizing environmental interferences, modification was made to *in vitro* collection and treatment methods based on the previous report [64]. Forty-cm-long one-year-old branches were separately collected from ‘RedY’ and ‘PinkY’ trees by randomly picking when the accumulated chilling hours (between 0 and 7.2°C) after defoliation exceeded 50 hours, and the branches were then bagged with wet towels to maintain at 4°C in the fridge for 400 h. Subsequently, the branches were placed in a 5% sucrose solution (with 5 cm depth) and cultivated in a growth chamber at 25°C, with a light intensity of 2000 lx, and a constant relative humidity of 70%, under a 16-hour light : 8-hour dark photoperiod. The basal ends of the branches were trimmed by 1 cm and the sucrose solution was replaced twice a week. After 12 days, budbreaks were observed, and the following floral developments were divided artificially into four stages based on their morphologic alterations from bud bursting to flower blooming. Because bud bursting and flower blooming of apical ends advances that of basal ends in branches, the anatomic dissection of floral buds of different developmental stages was performed every 3 days and tender petals were isolated from floral samples of the last two stages separately. Each petal sample was treated as a biological replicate, resulting in three replicates being obtained from three independent collection experiments for metabolic determination and transcriptome sequencing.

Qualitative and quantitative anthocyanins\flavonols detection of petal samples was performed in MetWare (Wuhan, China) based on the UPLC-ESI-MS/MS system, using the instruments (UPLC: ExionLC™ AD and 1.7 μm 2.1 mm*100 mm WatersACQUITY BEH C18 column; MS: Applied Biosystems 6500 Triple Quadrupole, QTRAP® 6500+ triple quadrupole–linear ion trap mass spectrometer equipped with electrospray ionization/ESI Turbo Ion-Spray interface operated in a positive-ion mode and controlled by Analyst 1.6.3 software) and following the procedures as described previously [65]. Transcriptome sequencing experiments were synchronously performed using the same materials as the anthocyanin\flavonols detection.

### Transcriptomic sequencing and data analyses

RNA was isolated using RNAprep Pure Plant Plus Kit (Tiangen Biotech, Beijing, China). The transcriptomic libraries were prepared by NEBNext Ultra RNA Library Prep Kit for Illumina #E7770 (NEB, MA, USA) and sequenced on Illumina platforms. The gene expression calculation and differentially expressed genes analysis followed the previously reported method [66, 67]. Briefly, the generated pair-end reads were filtered by fastp v1.2.3 ^61^ and aligned to the ‘RedY’ haplotype 1 genome using STAR 2.7.0e [68], then the BAM (Binary Alignment Map) result files were processed for computing gene expressional levels values by cufflink v2.2.1 [69] and the read counts of mapping genes were collected for differentially expressed genes analysis using ‘Deseq2’ (v1.40) R package [70]. Genes met the criteria of |log2FC| > 2 and *P* < 0.05 were selected as differentially expressed genes. In this study, fragments per kilobase of exon model per million mapped fragments (FPKM) values were chosen to evaluate expressional levels of specific genes. Gene ontology annotation and KEGG-based metabolic pathways enrichment analyses of differentially expressed genes were performed through KOBAS-I [71] specified on the *P. persica* species option.

### Candidate gene screening by protein and gene aligning

Fifty-three genes/proteins of flavonoid biosynthetic enzymes and regulatory factors were collected according to earlier validation studies [22, 26, 33, 34, 36, 39]. Their sequences were then aligned against the annotated gene/protein datasets of ‘RedY’ genome to search the identical genes/proteins of ‘RedY’ by local blast program (corresponding protein information and their encoding genes, as well as related referencing sources listed in Table S10, see online supplementary material). We calculated the gene expressional levels and investigate whether any sequence mutations occurred in 53 chosen genes/proteins. To ensure the reliability of calculate results obtained from comparative transcriptomics, we parallelly evaluated the expressional levels of 15 critical genes by qRT-PCR with earlier reported primers (Table S11, see online supplementary material), which were intended to made our work comparable with other studies.

Throughout alignment-base screening, a distinct structural variant in ‘RedY’-specific *Flavonol Synthetase* (*FLS*) was identified as a primary concern due to its prominent sequence variance. ‘RedY’-specific *FLS* harbored two insertional sequences in intron1 and exon2 comparing to previously identified *FLS*.

### Genomic DNA re-sequencing

Genomic DNA was extracted using DNAquick Plant System Kit (Tiangen Biotech, Beijing, China) and used for DNA-seq. Indexed libraries were constructed by NEB Next® Ultra™ DNA Library Prep Kit #E7370L (NEB, MA, USA) and sequenced using Illumina Novaseq according to the manufacturer’s protocols.

### Insertional variation and genotype detection of FLS in peach germplasms

Because two sequential insertions were found in intron1 and exon2 of ‘RedY’-specific *FLS*, we adopted a timesaving alignment method to investigate whether either or both insertions exist in other peach germplasms.

For insertion in intron1, we used two 60-bp genomic sequence to detect the insertion. 5’-TCTAACTACATTTTCTCATTTTAATTTATA was the identical sequence before the insert site on position. Thus, we aligned 60-bp TCTAACTACATTTTCTCATTTTAATTTATA-*GAGATATTTAGTGATATACCCATTTCTAGC* sequence (*GAGATATTTAGTGATATACCCATTTCTAGC* came from the following intronic insertion) to re-sequencing dataset of peach cultivars, based on (NCBI hosted) SRA blastn program, detecting sequence variability. Likewise, non-intronic-insert sequence TCTAACTACATTTTCTCATTTTAATTTATA-*TTTAATAAAATTCTATTTGTTTAATTTAAT* was also searched concurrently. Briefly, SRA archive data of peaches were converted to FASTA-format reads using sratoolkit 3.0.6, and then these pair reads were batch-aligned against the detect sequences by blastn program, following NCBI manuals. Either or both 60-bp nucleotide sequences were considered to exist in the equivalent locus only if 100% similarity with 60-bp length was verified based on SRA reads blast-hit results.

Equally, TTCTCGTCCCCAACGATGTCCAGGGCCTCCAG-GGCCTCCAG-*GCCTCCAGAGATGGCCGCT* (insert variation in exon2) and TTCTCGTCCCCAACGATGTCCAGGGCCTCCAG-*GCCTCCAGAGATG**GCCGCT* (non-insert variation in exon2) were respectively blasted to SRA-deposited peaches re-sequencing data by the same criteria to survey the insertional variation distribution.

We used 168 peach resequencing data from previous studies [18, 19, 72, 73] and re-sequenced 24 peaches (including ‘PinkY’, ‘RedY’ and 22 red-flower peaches), which were collected by author or were provided by China National Peach Germplasm Resource Nurseries/CNPGRN (Nanjing and Zhengzhou repositories), for *FLS* insertional variation and genotype detection. The petal colors of most collected peaches were manually checked using a professional illustrated handbook [72] that described the detailed information of peach resources preserved in CNPGRN, including source, taxonomy, characteristics and images of flowers and mature fruit, etc. The same research team had released these peaches’ resequencing data and published the illustrated handbook with precise characteristic description, therefore providing a reliable data source that was the optimal selection in this study. Rest peaches’ petal color were retrieved or provided by CNPGRN staff. Concrete details of peaches are listed in Table S12 (see online supplementary material).

### Genome-wide association studies (GWAS)

Genomic DNA structural variations (SVs) of 191 peaches (168 pink-flower and 23 red-flower germplasms above-mentioned) were called by the Sentieon package [74] with the haplotype 1 genome as the reference genome, then those appropriate SVs filtered by GATK4 [75] (with parameter QD < 2.0, FS > 200.0, SOR > 10.0, MQRankSum < −12.5, ReadPosRankSum < −8.0) were selected for analysis. The GWAS was performed using EMMAX [76] with general linear model based on SVs and phenotypic data. According to the genome-wide association result, we used fast variant effect predictor snpEff v4_3t [77] to annotate and investigate genes associated with the significant loci and the influences of structural variances.

### FLS prokaryotic expression and functional verification

Two types of Flavonol Synthetase (FLS) enzyme, one representing the previously reported FLS and the other representing the variant FLS identified in ‘RedY’, were amplified from ‘PinkY’ and ‘RedY’ cultivars, respectively, using reverse transcription polymerase chain reaction (RT-PCR). The amplified genes were then cloned into a prokaryotic expression vector, pET-28a(+), with a 6 × His tag using *Nde*I-*Xho*I endonuclease sites. The recombinant vectors were transformed into *Escherichia coli* BL21(DE3) using a heat shock method.

The expressed recombinant proteins with a molecular weight of 38 kDa were purified and harvested after overnight culture of the recombinant strains at 15°C in the presence of 0.2 mM Isopropyl β-D-Thiogalactoside (IPTG), a commonly used inducer for protein expression in *E. coli*. The correct expression of the recombinant proteins was verified by sodium dodecyl sulfate-polyacrylamide gel electrophoresis (SDS-PAGE).

Enzymatic activities of the purified recombinant FLS proteins were further evaluated by their ability to catalyze dihydrokaempferol substrates to generate kaempferol product, which was determined by high-performance liquid chromatography (HPLC) analyses (Waters E2695 system (Milford, MA) equipped with a Waters Sunfire 4.6 × 250 mm 5 μm C18 Column and 2998 photodiode array detector). 200 μL FLS enzymatic reaction system consisted of 0.1 M Tris/HCl (pH 7.4), 50 μM FeSO_4_, 0.1 mg/mL BSA, 2 mg/mL ascorbic acid, 1.5 mg/mL α-ketoglutarate, 0.5 mg/mL catalase, 50 μg/mL dihydrokaempferol, and 2 mg/mL recombinant FLS protein. Reaction ran at 30°C for 100 min under aerobic conditions, and terminated after the addition of 200 μL ethyl acetate stop solution. Subsequently, the reaction solution mixtures were vortexed and vacuum dried, then dissolved to methanol for HPLC analyses (mobile phases: 0.1% formic acid-water (A) and 0.1% formic acid-acetonitrile (B), gradient elute program: 90–50% A 0–7 min, 50% A constant 7–10 min, 50–0% A 10–15 min, 0–90% A 15–15.1 min, 90% A constant 15.1–16 min; flow rate 1 mL/min; column temperature 25°C; injection volume 10 μL; monitoring at 360 nm for product quantification). In our study, FLS enzyme activities were measured by the product generation. We defined 1 μg kaempferol product generation catalyzed by 1 mg recombinant protein within 100 min reaction time as 1 Unit FLS enzymatic activity.

## Supplementary Material

Web_Material_uhad210

## Data Availability

The raw sequencing data of current study were deposited in the Genome Sequence Archive (GSA, https://ngdc.cncb.ac.cn/gsa/) of the National Genomics Data Center (NGDC) / China National Center for Bioinformation (CNCB) under the following accession numbers: PacBio de novo Hifi sequencing of ‘RedY’: CRA009407; Hi-C sequencing of ‘RedY’: CRA009408; Illumina RNA-Seq paired end reads of ‘RedY’ diverse tissues: CRA009422; Illumina RNA-Seq paired end reads of ‘RedY’ and ‘PinkY’ petal at four developmental stages: CRA009421. The re-sequencing data of 24 peaches were publicly accessible at NCBI Sequence Read Archive under accession PRJNA951060. *P. persica* RedY whole genome v1.0 assembly and annotation are available in Genome Warehouse (GWH, https://ngdc.cncb.ac.cn/gwh/) at NGDC / CNCB under accession number GWHCBHS00000000.
